# Effects of multimorbidity patterns and socioeconomic status on catastrophic health expenditure of widowed older adults in China

**DOI:** 10.3389/fpubh.2023.1188248

**Published:** 2023-08-11

**Authors:** Zhen Wang, Zhi Zeng

**Affiliations:** ^1^School of Public Health, Hubei University of Medicine, Shiyan, China,; ^2^Center of Health Administration and Development Studies, Hubei University of Medicine, Shiyan, China

**Keywords:** catastrophic health expenditure, China, multimorbidity patterns, socioeconomic status, widowed older adults

## Abstract

**Background:**

The high multimorbidity and lower socioeconomic status (SES) of older adults, can lead to catastrophic health expenditures (CHEs) for older adults’ households. However, whether widowed older adults will bear such a financial burden has yet to be explored. The aim of this study was to investigate the influence of multimorbidity patterns and SES on CHE in Chinese widowed older adults.

**Methods:**

Data was obtained from the 2018 China Health and Retirement Longitudinal Study (CHARLS). This is a cross-sectional study. A total of 1,721 widowed participants aged 60 years and older were enrolled in the study. Latent class analysis was performed based on 14 self-reported chronic diseases to identify multimorbidity patterns. The logistic model and Tobit model were used to analyze the influence of multimorbidity patterns and SES on the incidence and intensity of CHE, respectively.

**Results:**

About 36.72% of widowed older adults generated CHE. The incidence and intensity of CHE were significantly higher in the cardiovascular class and multisystem class than in the minimal disease class in multimorbidity patterns (cardiovascular class, multisystem class, and minimal disease class). Among SES-related indicators (education, occupation and household *per capita* income), respondents with a middle school and above education level were more likely to generate CHE compared to those who were illiterate. Respondents who were in the unemployed group were more likely to generate CHE compared to agricultural workers. In addition, respondents aged 70–79 years old, geographically located in the east, having other medical insurance, or having fewer family members are more likely to generate CHE and have higher CHE intensity.

**Conclusion:**

Widowed older adults are at high risk for CHE, especially those in the cardiovascular and multisystem disease classes, and those with low SES. Several mainstream health insurances do not provide significant relief. In addition, attention should be paid to the high-risk characteristics associated with CHE. It is necessary to carry out the popularization of chronic disease knowledge, improve the medical insurance system and medical service level, and provide more policy preferences and social support to widowed older adults.

## Introduction

Catastrophic health expenditure (CHE) is generally defined as a household’s health expenditure as a percentage of total expenditure or income level reaching a threshold that has a catastrophic impact on the family’s financial situation and quality of life (1–3). The occurrence of CHE continues to increase worldwide. It has been demonstrated that the occurrence of CHE increased significantly in the 133 countries surveyed during the decade 2000 to 2010, regardless of which criteria was used to define CHE (4). This trend is much more pronounced in low- and middle-income countries, where health spending is growing at about 6% a year, 2% more than in high-income countries (5). Medical insurance systems in some low-income countries have achieved good results, such as China’s almost universal coverage (6), but due to the limited capital pool, it is difficult to fully protect the actual interests of the insured (7, 8). Studies have proved that the incidence of CHE in Chinese families is 13.9% (9) higher than some other countries, such as South Korea (10), Nepaland (11), and Bhutan (12). The proportion of OOP health expenditure in China’s total health expenditure is higher than the normal range defined by the WHO (13). It remains an important issue for China’s health authorities to provide more effective financial risk protection to its residents (14).

Multimorbidity refers to the occurrence of two or more chronic diseases in an individual at the same time (15), and it can share the same or different causes (16, 17), and has a significant negative impact on the health status, quality of life, and health expenditure of patients (18–21). Patients with multimorbidity have higher health expenditure than non-multimorbidity patients in developed countries, and already affect more than 60% of older adults (22, 23). Developing countries are not any exception. For example, nearly 70% of older adults in China suffer from multi-morbidity (24), and the medical complex service conditions required to treat multi-morbidity are a great challenge for the health care system (25, 26). Patients with different patterns of multimorbidity may have different care practices and health expenditures. Some scholars have used cluster analysis to cluster the information of chronic diseases of individuals (17, 27, 28), being aimed at better identify the potential categories of observed variables and conduct research on cognitive function, related outcomes, and related factors.

Contextual factors such as the physical characteristics of the environment, the sociocultural characteristics of the community and the accessibility of related services have some influence on health expenditure (29). When patients are under these adverse conditions, they may need to bear a greater risk of adverse health outcomes (30). Due to their lower socioeconomic status (SES), they have a lower economic level, and their money on medical and health services is likely to occupy a large proportion of their total household expenditure, thus generating CHE. In addition, the occurrence of CHE is associated with unequal socioeconomic status (e.g., employment, education, income), and subjects with CHE are more likely to be taken those with lower socioeconomic status (31, 32). Some SES factors, such as household income *per capita*, have been declared to have a significant impact on household CHE for older adults patients with chronic diseases. Equity in policies that reduce the incidence of CHE must be promoted by addressing socioeconomic factors that impact healthcare outcomes for older adults (33). The fairness of medical insurance design in China needs to be improved. For example, applying for the protection of the Medical Assistance program requires patients to have a certain level of health knowledge and be able to successfully fill out complex application forms, which disadvantage people of lower socioeconomic status (34, 35). It is therefore imperative to clarify differences in the equity of Medicare benefits and access to quality health services among patients of different socioeconomic status.

China holds the largest older adults population in the world (36). While the aging of the population is deepening, the widowed rate of older adults is also increasing. Depending upon the results of China’s seventh census, the number of older adults in China has reached 264 million, accounting for 18.70 percent of the total population. The number of widowed older adults has risen to 50 million, accounting for 18.9 percent of the total older adults population, an increase of nearly 2.6 million compared with 2010. The loss of a spouse is known as one of the most stressful events in human life (37), and its negative impact often involves the health status, economic level, quality of life and other aspects of the widowed person (38–41). Therefore, more attention should be paid to the economic burden of disease in widowed older adults, aiming to improve their quality of life in later life.

In this study, we aimed to evaluate multimorbidity patterns and the impact of SES on the incidence and intensity of CHE in widowed older adults in China. This is a cross-sectional study. The main challenge of this study is to identify the comorbidity pattern of widowed older adults in China and explore the influencing factors of CHE in widowed older adults. The main value and significance of this study is to provide a theoretical basis for the formulation of programs to alleviate the economic burden of diseases of widowed older adults, improve the quality of medical and health services, and perfect the medical insurance system, so as to promote the realization of healthy aging.

## Methods

### Data source

Data were obtained from the China Health and Retirement Longitudinal Study (CHARLS), a nationally representative longitudinal survey of Chinese adults aged 45 years and above (42). The baseline survey of CHARLS was undertaken in 2011, followed by four waves of surveys in 2013, 2015, and 2018. Electronic mapping software (GIS) was used to construct village-level sampling frame by mapping method, and multi-stage probability proportional to size (PPS) sampling was used. The survey covered 150 county-level units and 450 community-village level units in 28 provinces (autonomous regions and municipalities directly under the Central Government). The purpose of this study was to obtain relevant data on Chinese residents aged 45 and older, such as socio-demographic characteristics, family information, health status, medical insurance, work status, and medical service utilization of the respondents. CHARLS has received ethics approval from the Biomedical Ethics Review Board of Peking University (IRB0000105IRB 00001052–11015).

In this study, we used the data of CHARLS 2018, the fourth wave of its survey data, with a total of 19,816 respondents. Participants who met the required criteria (age 60 years and above, marital status as widowed, and complete information on chronic diseases and CHE related information) were included in the study, and 1,721 participants were finally included.

### Measurement of catastrophic health expenditure (dependent variable)

The dependent variables in this study were the incidence and intensity of CHE. Build on previous research (31, 43–45), we measured CHE as out-of-pocket (OOP) medical expenses equal to or exceeding 40% of the family’s ability to pay (CTP). We included annual non-food household consumption expenditure as a measure of household ability to pay (CTP) to avoid measurement biases that may be missed by poor households (31, 46).

Annual household health expenditure was regarded as out-of-pocket (OOP) medical expenditure. We used pooled binary variables to determine the presence or absence of CHE in a participant’s household:


$$CHE_{i}=\{\begin{array}{c} 1,\frac{OOP_{i}}{CTP_{i}}\geq z\\ 0,\frac{OOP_{i}}{CTP_{i}}<z \end{array}$$

Among them, $OOP_{i}$ represents annual household health expenditure. The $CTP_{i}$ represents annual non-food household consumption expenditures, with z being 40% of the disaster threshold.

The strength of CHE indicates the extent to which OOP payments affect family life (47). It is expressed as CTP’s OOP payment proportion minus the threshold, calculated as follows:


$$Intensity_{i}=\{\begin{array}{c} \frac{OOP_{i}}{CTP_{i}}-z,\frac{OOP_{i}}{CTP_{i}}\geq z\\ \;0,\frac{OOP_{i}}{CTP_{i}}<z \end{array}$$

Among them, $OOP_{i}$ represents the annual health expenditure cost of household i. $CTP_{i}$ is the non-food expenditure of household i and z is the catastrophe threshold of 40%.

### Identification of multimorbidity patterns (independent variable)

The CHARLS 2018 survey collected information on whether participants had been diagnosed with any of the following 14 chronic conditions: hypertension, dyslipidemia, diabetes, cancer or malignant tumor, chronic lung disease, liver disease, heart disease, stroke, kidney disease, stomach and other digestive disease, emotional and psychiatric problems, memory-related disease, arthritis or rheumatism, and asthma. All the diseases were treated as dichotomous variables. LCA was used to identify multimorbidity patterns and to identify the category to which participants belonged.

### SES indicators (independent variable)

SES measures used to represent the individual level include: education, occupation, and household *per capita* income. It has been shown that a higher level of education of an individual is associated with a lower frequency of use of medical services but a greater willingness to pay for medical services when needed (48, 49). Since the age of the subjects was not less than 60 years, we classified three levels of education: illiterate, primary school, middle school and above. The employment status and urban and rural residence of Chinese residents determine the type of medical insurance that individuals participate in. We categorized occupation types into four categories: agricultural work (at least 10 days of agricultural work in the past year and no other four categories of employment status were involved), non-agricultural work, retired (including early retirement, that is, people who quit before retirement age but enjoy the same benefits as normal retirement), and unemployed. Household *per capita* income is the average income of household members in the past 12 months.

### Covariates

Covariates included in this study were: gender (male and female), age group (60–69 years, 70–79 years and ≥ 80 years), residence (urban and rural), geographic location (Northeast, East, central and West), type of health insurance [urban employee basic medical insurance (UEBMI), urban resident basic medical insurance (URBMI), new rural cooperative medical insurance (NRCMI), other MIs and no medical insurance (MI)], any financial support from family members or relatives and friends (yes or no), provision of any financial support to family members or relatives and friends (yes or no), difficulties in activities of daily living (ADL: eating, bathing, dressing, toileting, going to bed, and urinating; yes or no), and number of family members.

### Introduction to logistic and Tobit model

Logistic regression model is one of the most important and widely used nonlinear statistical models to analyze dichotomous variables. In this model, the dependent variable is binary (y = 0 or y = 1), and the outcome variable has a nonlinear relationship with the independent variables. Tobit regression model is a kind of linear regression model, which is characterized by the truncation phenomenon of its dependent variable. Truncation means that some observations are not observed, that is, they are limited to a certain range of values. Tobit regression model can transform the truncated data into a probability model, and then perform statistical analysis on the truncated data.

### Statistical analysis

Multimorbidity patterns as well as category affiliation of individuals were determined by performing LCA on chronic disease information from 1,721 participants. The use of LCA to identify case subclasses in multivariate categorical data can achieve excellent results (50). The statistical data and clinical significance of the various schemes were taken into account when selecting the best grouping of multimorbidity patterns.

Descriptive statistics were performed for each variable using the frequency distribution, and the differences between the two groups were compared using the chi-square test. Logistic regression model was used to analyze the influence of multimorbidity patterns and SES on the incidence of CHE. Tobit regression model was used to analyze the influence of multimorbidity patterns and SES on CHE intensity. CHE intensity was a continuous variable of no less than 0, and more than 60% of the participants had CHE intensity equal to 0, so the Tobit regression model applicable to the truncated data was utilized (51).

LCA was performed using Mplus version 8.3, and Stata version 16.0 was used for all statistical analyses. Two-sided *p* values of less than 0.05 were envisaged for indicate statistical significance.

## Results

### Sample characteristics

Of 1,721 participants, 73.39% were female. Most were in the 70–79 age group (39.45%), and more than four out of five (81.12%) lived in rural areas. About 6.16, 28.82, 28.82 and 36.20% of the subjects resided in northeastern, eastern, central and western China, respectively. Most participants (64.85%) were enrolled in NCRCMS. 89.77% received financial support from family members, and 50.49% provided financial support to family members. Nearly one-fifth (19.99%) of the subjects had difficulty in ADL. A total of 632 participants (36.72%) had CHE. The mean intensity of CHE was 0.108 with a standard deviation of 0.182 (Table 1).

**Table 1:** Incidence of catastrophic health expenditure (CHE) by sociodemographic characteristics (*n* = 1,721).

Characteristics	All	With CHE (*n* = 632)	Without CHE (*n* = 1,089)	χ^2^/t	*p*
	*n* (%)	*n* (%)	*n* (%)		
Multimorbidity patterns				43.003	<0.001
Cardiovascular class	319 (18.54)	155 (48.59)	164 (51.41)		
Multisystem class	288 (16.73)	130 (45.14)	158 (54.86)		
Minimal disease class	1,114 (64.73)	347 (31.15)	767 (68.85)		
Socioeconomic status (SES)					
Education				8.851	0.012
Illiterate	797 (46.31)	312 (39.15)	485 (60.85)		
Primary school	692 (40.21)	254 (36.71)	438 (63.29)		
Middle school and above	232 (13.48)	66 (28.45)	166 (71.55)		
Occupation				14.164	0.003
Agricultural work	438 (25.45)	134 (30.59)	304 (69.41)		
Non-agricultural work	100 (5.81)	33 (33.00)	67 (67.00)		
Retired/receded	233 (13.54)	80 (34.33)	153 (65.67)		
Unemployed	950 (55.20)	385 (40.53)	565 (59.47)		
Household *per capita* income				24.778	<0.001
Lowest 20%	308 (17.90)	95 (30.84)	213 (69.16)		
Lower 20%	310 (18.01)	129 (41.61)	181 (58.39)		
Middle 20%	308 (17.90)	140 (45.45)	168 (54.55)		
Higher 20%	310 (18.01)	110 (35.48)	200 (64.52)		
Highest 20%	310 (18.01)	92 (29.68)	218 (70.32)		
Missing	175 (10.17)	66 (37.71)	109 (62.29)		
Gender				7.492	0.006
Male	458 (26.61)	144 (31.44)	314 (68.56)		
Female	1,263 (73.39)	488 (38.64)	775 (61.36)		
Age				20.569	<0.001
60–69 years	565 (32.83)	166 (29.38)	399 (70.62)		
70–79 years	679 (39.45)	282 (41.53)	397 (58.47)		
≥80 years	477 (27.72)	184 (38.57)	293 (61.43)		
Residency				0.467	0.494
Rural	1,396 (81.12)	518 (37.11)	878 (62.89)		
Urban	325 (18.88)	114 (35.08)	211 (64.92)		
Geographic location				10.936	0.012
Northeast	106 (6.16)	40 (37.74)	66 (62.26)		
East	496 (28.82)	209 (42.14)	287 (57.86)		
Central	496 (28.82)	180 (36.29)	316 (63.71)		
West	623 (36.20)	203 (32.58)	420 (67.42)		
Health insurance				5.950	0.203
Urban employee basic medical insurance (UEBMI)	177 (10.28)	57 (32.20)	120 (67.80)		
Urban resident basic medical insurance (URBMI)	73 (4.24)	27 (36.99)	46 (63.01)		
New rural cooperative medical insurance (NRCMI)	1,116 (64.85)	415 (37.19)	701 (62.81)		
Other MIs	261 (15.17)	106 (40.61)	155 (59.39)		
No medical insurance	94 (5.46)	27 (28.72)	67 (71.28)		
Receive any financial support				2.030	0.154
No	176 (10.23)	56 (31.82)	120 (68.18)		
Yes	1,545 (89.77)	576 (37.28)	969 (62.72)		
Provide any financial support				3.285	0.070
No	852 (49.51)	331 (38.85)	521 (61.15)		
Yes	869 (50.49)	301 (34.64)	568 (65.36)		
Any ADL difficulty				3.841	0.050
No	1,377 (80.01)	490 (35.58)	887 (64.42)		
Yes	344 (19.99)	142 (41.28)	202 (58.72)		
Number of family members				7.067	<0.001
Mean ± SD (standard deviation)	1.969 ± 1.458	1.647 ± 1.243	2.155 ± 1.540		
Intensity of CHE					
Mean ± SD (standard deviation)	0.108 ± 0.182	0.294 ± 0.187	0		

**Figure 1:**
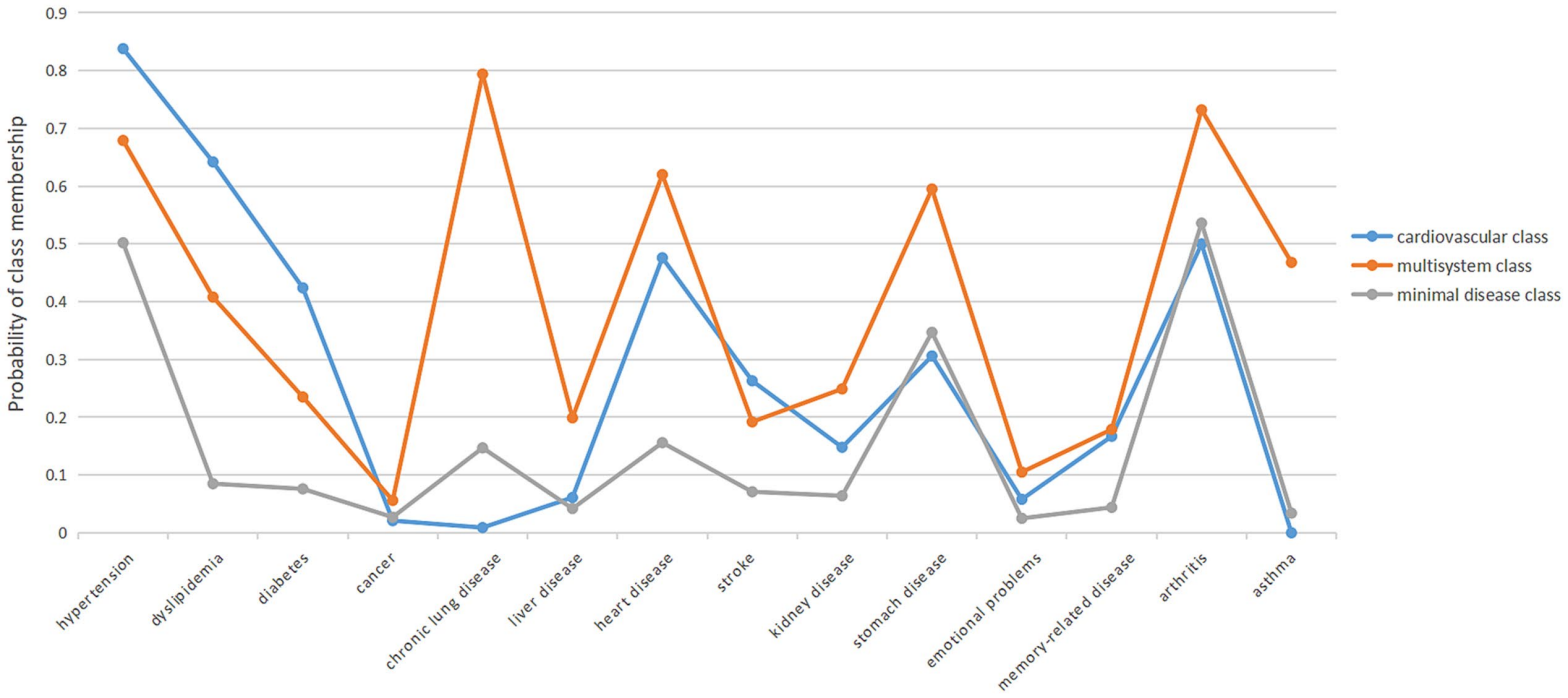
Three-class model of multimorbidity patterns among widowed older adults.

In identifying multimorbidity patterns, we examined class 2–5 models by LCA. The three-level model appeared to be the best-fitting model (Table 2) and showed the most reasonable clinical interpretability. Three patterns of multimorbidity were named cardiovascular class, multisystem class, and minimal disease class on the basis of the item-response probability as compared with the population average (Figure 1). The minimal disease class included relatively healthy participants with a low prevalence of 14 chronic diseases. The majority (64.73%) of the participants belonged to this pattern. The cardiovascular class includes individuals with a higher prevalence of hypertension, diabetes, heart attack, stroke, dyslipidemia, etc. About 18.54% of the participants were in the cardiovascular class. Participants in the multisystem class had a very high prevalence of 14 chronic conditions. Approximately 16.73% of the participants were in the multisystem class. In addition, the average number of chronic diseases in minimal disease class ranges is 1.781, the average number of chronic diseases in the cardiovascular class ranges is 4.282 and the average number of chronic diseases in the multisystem class ranges is 5.767.

**Table 2:** Model comparison of latent class analysis ft. in multimorbidity patterns.

No. classes	AIC	BIC	aBIC	Entropy	LMR
2	20890.036	21048.105	20955.975	0.619	0.0060
3	20678.855	20918.684	20778.901	0.651	0.0300
4	20579.601	20901.190	20713.753	0.575	0.0639
5	20536.232	20939.581	20704.491	0.585	0.0848

Regarding the educational attainment of SES. Nearly half (46.31%) of the participants were illiterate. In terms of occupation type, more than half (55.20%) of the participants were unemployed. Participants who did not provide information on household income *per capita* were grouped into a single category. Whereas the others were divided into five equal groups from lowest to highest (Table 1).

### Comparison of differences between the two groups

Gender (*p* = 0.006), age (*p* < 0.001), geographic location (*p* = 0.012), multimorbidity patterns (*p* < 0.001), education (*p* = 0.012), occupation (*p* = 0.003), household *per capita* income (*p* < 0.001) and number of family members (*p* < 0.001) were significant differences between the group with CHE and the group without CHE (Table 1). Among them, women have a higher incidence of CHE (38.64%). Participants with 70–79 years are more likely to develop CHE than other age groups (41.53%). Participants in the east are more likely to develop CHE than those in other regions (42.12%). Participants with fewer family members have a higher incidence of CHE. Participants in the cardiovascular disease group were more likely to develop CHE than those in other groups (48.59%). Illiterates were more likely to develop CHE than those with other levels of education (39.15%). Unemployed participants were more likely to develop CHE than those with other occupational types (40.53%). Participants with middle income were more likely to experience CHE than those with other incomes (45.45%).

### Logistic regression results

We included the core independent variables, multimorbidity patterns and SES, in Model 1 and then further included all covariates in Model 2. The risk of CHE in the multi-system class and the cardiovascular class was significantly higher than that in the minimal disease class (*p* < 0.001). Specifically, subjects in the cardiovascular class were most likely to generate CHE (Model 1: OR = 2.134, 95% CI = 1.642–2.774; Model 2: OR = 2.025, 95%CI = 1.543–2.657), followed by multisystem class (Model 1: OR = 1.774, 95% CI = 1.355–2.323; Model 2: OR = 1.730, 95% CI = 1.308–2.290). In SES, education level of middle school and above was a protective factor for CHE (Model 1: OR = 0.621, 95% CI = 0.435–0.885; Model 2: OR = 0.669, 95% CI: 0.454–0.984), and unemployed occupation type presented a risk factor for CHE (Model 1: OR = 1.500, 95% CI = 1.170–1.923; Model 2: OR = 1.408, 95% CI: 1.075–1.844). In addition, the incidence of CHE was higher in participants aged 70 to 79 years, geographically located in the East, with other insurance type, or with a relatively small number of family members (*p* < 0.05, 0.01, or 0.001; Table 3).

**Table 3:** Predictors of incidence of catastrophic health expenditure (CHE): results of logistic regression model.

	Model 1		Model 2	
	Odds ratio	95% CI	Odds ratio	95% CI
**Multimorbidity patterns (Ref. minimal disease class)**				
Cardiovascular class	2.134^***^	[1.642, 2.774]	2.025^***^	[1.543, 2.657]
Multisystem class	1.774^***^	[1.355, 2.323]	1.730^***^	[1.308, 2.290]
**Socioeconomic status (SES)**				
**Education (Ref. illiterate)**				
Primary school	0.926	[0.744, 1.152]	0.957	[0.753, 1.215]
Middle school and above	0.621^**^	[0.435, 0.885]	0.669^*^	[0.454, 0.984]
**Occupation (ref: agricultural work)**				
Non-agricultural work	1.403	[0.862, 2.282]	1.369	[0.823, 2.277]
Retired/receded	1.618^*^	[1.075, 2.434]	1.365	[0.839, 2.221]
Unemployed	1.500^**^	[1.170, 1.923]	1.408^**^	[1.075, 1.844]
**Household *per capita* income (ref: the lowest 20%)**				
Lower 20%	1.626^**^	[1.159, 2.281]	1.102	[0.762, 1.594]
Middle 20%	1.890^***^	[1.350, 2.647]	1.293	[0.898, 1.862]
Higher 20%	1.210	[0.855, 1.713]	1.074	[0.745, 1.547]
Highest 20%	0.901	[0.605, 1.343]	0.644	[0.408, 1.015]
Missing	1.439	[0.966, 2.145]	1.039	[0.678, 1.591]
**Gender (Ref. male)**				
Female			1.241	[0.961, 1.602]
**Age (Ref. 60–69 years)**				
70–79 years			1.438^**^	[1.113, 1.857]
≥80 years			1.259	[0.933, 1.698]
**Residency (Ref. rural)**				
Urban			1.098	[0.672, 1.795]
**Geographic location (Ref. west)**				
Northeast			1.248	[0.791, 1.967]
East			1.394^*^	[1.064, 1.827]
Central			1.099	[0.843, 1.434]
**Health insurance (Ref. no medical insurance)**				
Urban employee basic medical insurance (UEBMI)			1.655	[0.798, 3.430]
Urban resident basic medical insurance (URBMI)			1.494	[0.675, 3.306]
New rural cooperative medical insurance (NRCMI)			1.622	[0.994, 2.645]
Other MIs			1.809^*^	[1.052, 3.111]
**Receive any financial support (Ref. no)**				
Yes			1.048	[0.730, 1.504]
**Provide any financial support (Ref. no)**				
Yes			0.875	[0.702, 1.092]
**Any ADL difficulty (Ref. no)**				
Yes			1.135	[0.868, 1.483]
Number of family members			0.761^***^	[0.694, 0.833]
Constant	0.279^***^		0.232^***^	

^*^p < 0.05, ^**^p < 0.01, ^***^p < 0.001.

### Tobit regression results

Both Model 1 and Model 2 showed that the intensity of CHE of the cardiovascular class (Model 1: ME = 0.060, *p* < 0.001; Model 2: ME = 0.051, *p* < 0.001) and multi-system cluster (Model 1: ME = 0.057, *p* < 0.001; Model 2: ME = 0.053, *p* < 0.001) were significantly higher than that of the minimal disease group. In SES, Education level of middle school and above (Model 1: ME = −0.040, *p* < 0.01; Model 2: ME = −0.030, *p* < 0.05), and the unemployed (Model 1: ME = 0.031, *p* < 0.01; Model 2: ME = 0.024, *p* < 0.05) had a higher CHE intensity. In addition, CHE intensity was significantly higher among participants who aged 70–79 years, geographically located in the East, had UEBMI, NRCMI or other MIs, or who had a relatively small number of family members (*p* < 0.05, 0.01, or 0.001; Table 4).

**Table 4:** Predictors of increasing the intensity of catastrophic health expenditure (CHE): results of Tobit regression model.

	Model 1		Model 2	
	Marginal effect	95% CI	Marginal effect	95% CI
**Multimorbidity patterns (Ref. minimal disease class)**				
Cardiovascular class	0.060^***^	[0.034, 0.086]	0.051^***^	[0.027, 0.076]
Multisystem class	0.057^***^	[0.030, 0.083]	0.053^***^	[0.027, 0.079]
**Socioeconomic status (SES)**				
**Education (Ref. illiterate)**				
Primary school	−0.014	[−0.033, 0.006]	−0.008	[−0.274, 0.012]
Middle school and above	−0.040^**^	[−0.066, −0.014]	−0.030^*^	[−0.058, −0.002]
**Occupation (ref: agricultural work)**				
Non-agricultural work	0.031	[−0.011, 0.073]	0.030	[−0.0133, 0.074]
Retired/receded	0.030	[−0.005, 0.065]	0.007	[−0.030, 0.044]
Unemployed	0.031^**^	[0.012, 0.051]	0.024^*^	[0.003, 0.045]
**Household *per capita* income (ref: the lowest 20%)**				
Lower 20%	0.046^**^	[0.017, 0.075]	0.010	[−0.021, 0.041]
Middle 20%	0.064^***^	[0.034, 0.094]	0.028	[−0.004, 0.059]
Higher 20%	0.014	[−0.013, 0.040]	0.004	[−0.026, 0.034]
Highest 20%	−0.008	[−0.036, 0.020]	−0.038^*^	[−0.070, −0.006]
Missing	0.039^*^	[0.005, 0.074]	0.009	[−0.026, 0.045]
**Gender (Ref. male)**				
Female			0.029^**^	[0.009, 0.048]
**Age (Ref. 60–69 years)**				
70–79 years			0.033^**^	[0.013, 0.053]
≥80 years			0.027	[−0.001, 0.046]
**Residency (Ref. rural)**				
Urban			0.009	[−0.033, 0.051]
**Geographic location (Ref. west)**				
Northeast			0.028	[−0.011, 0.068]
East			0.031^**^	[0.009, 0.054]
Central			0.007	[−0.014, 0.027]
**Health insurance (Ref. no medical insurance)**				
Urban employee basic medical insurance (UEBMI)			0.062^*^	[0.007, 0.118]
Urban resident basic medical insurance (URBMI)			0.039	[−0.017, 0.095]
New rural cooperative medical insurance (NRCMI)			0.048^**^	[0.019, 0.076]
Other MIs			0.052^**^	[0.017, 0.086]
**Receive any financial support (Ref. no)**				
Yes			−0.003	[−0.033, 0.027]
**Provide any financial support (Ref. no)**				
Yes			−0.014	[−0.032, 0.004]
**Any ADL difficulty (Ref. no)**				
Yes			0.014	[−0.009, 0.036]
Number of family members			−0.025^***^	[−0.032, −0.018]

^*^p < 0.05, ^**^p < 0.01, ^***^p < 0.001.

### Additional information tables based on relevant results

According to the logistic and Tobit regression results, we compared the characteristics of the target population with the regional characteristics. This statistical information serves to elucidate the relative position of the selected sample within the broader population spectrum, in order to provide some reference for the formulation of relevant policies (Table 5).

**Table 5:** Comparison between target population characteristics and regional characteristics.

Characteristics	All	Northeast (*n* = 106)	East (*n* = 496)	Central (*n* = 496)	West (*n* = 623)
		*n* (%)	*n* (%)	*n* (%)	*n* (%)
**Multimorbidity patterns**					
Cardiovascular class	319	24 (7.52)	107 (33.54)	106 (33.23)	82 (25.71)
Multisystem class	288	13 (4.51)	65 (22.57)	77 (26.74)	133 (46.18)
**Education**					
Middle school and above	232	27 (11.64)	61 (26.29)	85 (36.64)	59 (25.43)
**Occupation**					
Unemployed	950	53(5.58)	275 (28.95)	263 (27.68)	359 (37.79)
**Age**					
70–79 years	679	41 (6.04)	175 (25.77)	204 (30.04)	259 (38.14)
**Health insurance**					
Other MIs	261	7 (2.68)	117 (44.83)	53 (20.31)	84 (32.18)
**Number of family members**					
Mean ± SD	1,721	1.774 ± 1.165	1.794 ± 1.363	1.821 ± 1.355	2.258 ± 1.606

## Discussion

This study used nationally representative CHARLS 2018 data to determine the incidence and intensity of CHE in widowed older adults in China, aiming to assess the status of multimorbidity patterns and SES influence on it. The results of this study have implications for clinical practice guidance and health policy formulation.

In this study, the incidence of CHE in widowed older adults was 36.72%, which was higher than 31.5% obtained by the study also using CHARLS 2018 data (52), and the intensity of CHE was 0.108, which was higher than the results of previous studies that did not fully focus on widowed older adults (53). This may be because the subjects of these two studies were older adults and adults, respectively, while this study narrowed the scope for older adults with widowed marital status. In addition, among the 1,721 participants in this study, 1,263 were female, accounting for about 73.39%, and 1,396 were rural residents, accounting for about 81.12%. China’s traditional family model, in which men outside the home, women inside, often still exists in these aging people, especially in rural areas (54). The death of the husband cuts off the main source of family income and reduces the economic level of widowed female older adults, which increases the possibility of CHE (39, 55–58). In addition, losing the care and companionship of a spouse is not conducive to physical and mental health, which may aggravate chronic diseases or lead to depression. In addition, China’s health resources tend to be in economically developed areas, and patients living in rural areas are therefore difficult to obtain the same level of health services (59). This may cause the deterioration of chronic diseases in rural patients with lower economic levels to be difficult to alleviate, thus making CHE occur through the continuous increase in health expenditure.

In this study, patterns of multimorbidity were identified from Chinese widowed older adults population, including the cardiovascular class, the multisystem class, and the minimal disease class. Among them, cardiovascular diseases are the biggest risk factors for CHE in subjects, followed by subjects with multi-system diseases, and they are also significant factors for the increase of CHE intensity. Studies have shown that the proportion of CHE in older adults with cardiovascular diseases in China exceeds that in developed countries, and the risk tolerance for medical payment is lower than the average level in China (60). Individuals with multiple chronic diseases are more apt to generate CHE (24). On the one hand, cardiovascular disease often requires long-term drug treatment and is a major contributor to the global economic burden of chronic diseases (61). In addition, cardiovascular diseases (such as hypertension, diabetes, and heart attacks) are associated with greater distress financing, leading households to be more inclined to borrow money or sell their property to be paid for related health costs (33, 62). On the other hand, expenditure on health care was positively correlated with the number of chronic diseases (33, 63–65), with participants in the multisystem class having higher expenditure on health care. Considering the economic level, mental status and other factors of widowed older adults, the families of patients in the cardiovascular class and the multisystem class are more likely to generate CHE and show higher CHE intensity values.

The present study found that the SES of widowed older adults people also affected the incidence and intensity of CHE in their families to some extent. There are related studies that also prove that CHE is related to SES (53, 66, 71). In this study, in terms of educational attainment, participants with middle school and above education had a lower probability of developing CHE. It may be because older adults with higher education level have stronger awareness of risk prevention, can effectively prevent the occurrence of chronic diseases (48), reduce their own economic burden of disease, and thus have a lower incidence of CHE. In terms of occupation type, CHE was more likely to occur in households of participants who were unemployed. It has been found that the families of unemployed older adults are more likely to generate CHE compared with agricultural workers, being consistent with the results of this study (67). The unemployed widowed older adults may have lost all economic sources. If there are no other family members in the family, the disease burden will bring significant negative effects on their economic level and quality of life, thus causing the occurrence of CHE. In terms of the number of family members, the higher the number, that is, the larger the household size, the lower the probability of CHE generation in older adults households. Similar studies have found that the family size of cancer patients is negatively correlated with the incidence of CHE (66), which is generally consistent with the results of this study. This may be due to the fact that more family members have more financial sources, daily care and spiritual support, and can effectively prevent and alleviate chronic diseases, so their incidence of CHE is lower.

This study showed that widowed older adults people aged 70–79 years, geographically located in the eastern region, and with other types of medical insurance were more likely to generate CHE, and the intensity of CHE is higher. There are regional differences in the allocation of medical and health resources in China, and the eastern regions with relatively developed economies have higher access to high-quality medical and health services (68). However, due to the low accessibility of medical and health services in other areas, it is therefore difficult to meet the medical service needs of patients, so that patients tend to produce a lower OOP medical expenditure (69, 70). China’s current medical insurance system has lowered the threshold for vulnerable groups to use medical and health services. However, it has not effectively alleviated the economic burden of disease of widowed older adults. When vulnerable groups can enter the threshold of medical and health services, OOP medical expenditure is generated. Even if this expenditure is relatively small, it has occupied a large proportion of the total family expenditure for them, which is likely to result in the occurrence of CHE and even produce high CHE intensity. It can be seen that China’s medical insurance system still have the shortcomings of insufficient policy protection in alleviating the economic burden of diseases in widowed older adults group.

### Limitations and strengths

This study has certain limitations. First of all, the information on income, expenditure and chronic diseases in CHARLS data are self-reported, which may affect study results due to recall bias or undiagnosed chronic diseases. Second, the database used in this study did not include a large enough number of chronic diseases, which may affect the classification results of multimorbidity patterns. Third, the perspectives considered in this study are limited, and factors such as depression assessment and social capital can be incorporated into future studies. Fourth, this study utilized a cross-sectional design, and the conclusions are not causal.

This study also has certain strengths. First, LCA was used to identify the multimorbidity patterns of Chinese widowed older adults, which can provide a reference for the comprehensive treatment plan in clinical practice. Second, there are two indicators to measure CHE in this study, and logistic and Tobit regression are used to analyze the results separately, which makes the results robust. Thirdly, this study found out the characteristics of people who are more likely to develop CHE, which provides a basis for the government to formulate relevant plans to resist CHE risk.

## Conclusion

According to nationally representative CHARLS 2018 data, we found that the incidence and intensity of CHE in widowed older adults were 36.72% and 0.108, respectively. Regarding multimorbidity patterns, we found that the incidence and intensity of CHE were higher in both the cardiovascular class and multisystem class than in the minimal disease class. Regarding SES, we found that the incidence and intensity of CHE were lower in the group with higher education level than in the illiterate group. The incidence and intensity of CHE were higher in unemployed than in agricultural workers. In addition, several mainstream medical insurance types did not effectively reduce the incidence and intensity of CHE, and those who chose other medical insurance had higher incidence and intensity of CHE than those who did not have medical insurance. Therefore, firstly, it is proposed to carry out the popularization of chronic disease knowledge on a large scale to prevent the occurrence of multimorbidity or slow down the deterioration of existing chronic diseases. Second, the amelioration and improvement of the medical insurance system and medical service institutions should increase the benefits to widowed older adults group, such as providing specific medical assistance plans for widowed older adults with cardiovascular diseases or multisystem diseases, and reasonably adjusting the detailed classification and payment scheme of the beneficiaries of medical insurance. Third, China’s health sector reform has achieved some results, but protecting vulnerable populations from health care-related poverty remains an ongoing challenge. Finally, relevant policies should also pay extra attention to groups of widowed older adults who have at least one of the following characteristics: the age range is 70–79 years, the geographical area belongs to the east, and the number of family members is small.

## Data availability statement

The original contributions presented in the study are included in the article/supplementary material, further inquiries can be directed to the corresponding author.

## Ethics statement

The studies involving human participants were reviewed and approved by the Biomedical Ethics Review Committee of Peking University (IRB0000105IRB00001052–11015). The patients/participants provided their written informed consent to participate in this study.

## Author contributions

ZW conceived the idea, analyzed the data, and wrote the manuscript. ZZ provided advice on research methods and discussion of the manuscript. All authors contributed to the article and approved the submitted version.

## Funding

The research was supported by supported by Key Research Center for Humanities and Social Sciences in Hubei Province (2022ZD002; Hubei university of Medicine) and Humanity and Social Science Youth Foundation of Ministry of Education of China (17YJCZH015).

## Conflict of interest

The authors declare that the research was conducted in the absence of any commercial or financial relationships that could be construed as a potential conflict of interest.

## Publisher’s note

All claims expressed in this article are solely those of the authors and do not necessarily represent those of their affiliated organizations, or those of the publisher, the editors and the reviewers. Any product that may be evaluated in this article, or claim that may be made by its manufacturer, is not guaranteed or endorsed by the publisher.
